# Adoption of evidence-based global policies at the national level: intermittent preventive treatment for malaria in pregnancy and first trimester treatment in Kenya, Malawi, Mali and The Gambia

**DOI:** 10.1093/heapol/czaa132

**Published:** 2020-11-12

**Authors:** Jayne Webster, Jenna Hoyt, Samba Diarra, Lucinda Manda-Taylor, George Okoth, Jane Achan, Ludovica Ghilardi, Umberto D’Alessandro, Mwayi Madanista, Simon Kariuki, Kassoum Kayentao, Jenny Hill

**Affiliations:** 1 Infectious and Tropical Diseases, London School of Hygiene and Tropical Medicine, Keppel St., London WC1E 7HT, UK; 2 Department of Clinical Sciences, Liverpool School of Tropical Medicine, Pembroke Place, Liverpool L3 5QA, UK; 3 Malaria Research and Training Centre, University of Sciences, Techniques, and Technologies of Bamako, Bamako BP: 1805, Mali; 4 School of Public Health and Family Medicine, College of Medicine, University of Malawi, Private Bag 360, Chichiri, Blantyre 3, Malawi; 5 Kenya Medical Research Institute/Centre for Global Health Research, Off Kisumu-Busia Road, PO Box 1578-4100 Kisumu, Kenya; 6 Medical Research Council Unit, The Gambia at the London School of Hygiene and Tropical Medicine, Atlantic Boulevard, Fajara, The Gambia

**Keywords:** Policy, malaria

## Abstract

In 2012, the World Health Organization (WHO) updated its policy on intermittent preventive treatment in pregnancy with sulphadoxine–pyrimethamine (IPTp-SP). A global recommendation to revise the WHO policy on the treatment of malaria in the first trimester is under review. We conducted a retrospective study of the national policy adoption process for revised IPTp-SP dosing in four sub-Saharan African countries. Alongside this retrospective study, we conducted a prospective policy adoption study of treatment of first trimester malaria with artemisinin combination therapies (ACTs). A document review informed development and interpretation of stakeholder interviews. An analytical framework was used to analyse data exploring stakeholder perceptions of the policies from 47 in-depth interviews with a purposively selected range of national level stakeholders. National policy adoption processes were categorized into four stages: (1) identify policy need; (2) review the evidence; (3) consult stakeholders and (4) endorse and draft policy. Actors at each stage were identified with the roles of evidence generation; technical advice; consultative and statutory endorsement. Adoption of the revised IPTp-SP policy was perceived to be based on strong evidence, support from WHO, consensus from stakeholders; and followed these stages. Poor tolerability of quinine was highlighted as a strong reason for a potential change in treatment policy. However, the evidence on safety of ACTs in the first trimester was considered weak. For some, trust in WHO was such that the anticipated announcement on the change in policy would allay these fears. For others, local evidence would first need to be generated to support a change in treatment policy. A national policy change from quinine to ACTs for the treatment of first trimester malaria will be less straightforward than experienced with increasing the IPTp dosing regimen despite following the same policy processes. Strong leadership will be needed for consultation and consensus building at national level.

Key Messages

- National policy decision-makers are responsible for the translation of global policies to national policies and their implementation. There is a lack of understanding of the variability in policy processes and factors influencing this.
- Our retrospective study of a prevention policy and prospective analysis of a treatment policy, found that the main drivers of policy adoption were: the methodological quality of the research, the relevance of the data to the local context and its prospects for effective implementation. Trust in WHO policy processes could override these factors.
- These findings contribute to gaps in understanding the translation of global to national policies.

## Introduction

Malaria in pregnancy (MiP) causes adverse effects for both the mother and baby including increased risk of maternal anaemia, low birthweight and prematurity (Desai *et al.*, 2007). The clinical effects of MiP depend on the intensity of malaria transmission, the parasite species and the level of immunity in pregnant women. There are three main strategies for prevention and control of MiP in sub-Saharan Africa (SSA), namely Long-Lasting Insecticidal Nets, intermittent preventive treatment with sulphadoxine–pyrimethamine (IPTp-SP) and effective case management of clinical malaria and anaemia (WHO Regional Office for Africa, 2004). IPTp-SP is the delivery of a treatment dose of the antimalarial drug SP given at prespecified times for the prevention of malaria, regardless of the presence of symptoms or confirmed malaria infection. This study addresses policies on both IPTp-SP and effective case management of first trimester malaria.

In terms of IPTp-SP, reports of increasing resistance to SP in parts of SSA (Chico *et al.*, 2015) (Desai *et al.*, 2017) have led to questioning of the continued efficacy of IPTp-SP among malaria control programmes. IPTp-SP was withdrawn in Rwanda in 2008 [Ministere de la Santé (MINISANTE), Institut National de la Statistique au Rwanda (INSR), 2009] and alternative drugs for IPTp (Desai *et al.*, 2015) and alternative strategies, primarily intermittent screening and treatment (Tagbor *et al.*, 2015), have been investigated in other countries. Findings of a meta-analysis of two vs three or more doses of IPTp with SP (Kayentao *et al.*, 2013) were presented at WHO’s Evidence Review Group (WHO-ERG) on MiP in July 2012 who, in turn, presented their recommendations to WHO’s Malaria Policy Advisory Committee (MPAC) in September 2012 (WHO Malaria Policy Advisory Committee, 2012). This led WHO to update its original two-dose IPTp policy to ‘a dose of SP at every ANC visit in the second and third trimester, at least one month apart’. The policy was subsequently communicated to WHO’s African Member States (WHO Global Malaria Programme, 2013) and several countries have now ratified the updated policy and/or begun implementation (Henry *et al.*, 2018).

For first trimester treatment of MiP, WHO recommends oral quinine (with or without clindamycin) for the case management of uncomplicated malaria in the first trimester, and artemisinin combination therapies (ACTs) or quinine or artesunate (AS) plus clindamycin in the second and third trimesters (WHO, 2010a). A recent systematic review of case management practices among healthcare providers revealed poor adherence to treatment guidelines by trimester across a range of countries in SSA (Hill *et al.*, 2014). Whilst ACTs are the first-line treatment for malaria in both children and adults across SSA, WHO does not currently recommend use of the artemisinin class of compounds in the first trimester of pregnancy because of insufficient safety data in early pregnancy in humans (Dellicour *et al.*, 2007; Ward *et al.*, 2007), unless this is the only treatment immediately available (WHO, 2010a). Yet systematic prescription of ACTs in the first trimester has been reported in several African countries (Hill *et al.*, 2014; Riley *et al.*, 2016). Furthermore, drugs that are no longer recommended in national treatment policies, such as SP (recommended for IPTp only) and chloroquine (CQ) (because of known high levels of parasite resistance to CQ) (Bloland, 2001), continue to be used. In addition, the use of artemisinin monotherapies is a major threat to the development of artemisinin resistance in the Africa region, as has occurred in parts of southeast Asia (WHO, 2010b; Duong *et al.*, 2012).

In 2015, a meta-analysis of observational studies on the risk of adverse pregnancy outcomes associated with first-trimester treatment with artemisinin derivatives and quinine (Dellicour *et al.*, 2017) was presented to a WHO-ERG (WHO, 2015). The meta-analysis concluded that compared to quinine, artemisinin treatment in the first trimester was not associated with an increased risk of miscarriage or stillbirth. The results were also reviewed by WHO’s MPAC in 2015 (WHO Malaria Policy Advisory Committee, 2016b) which recommended the review of the WHO Guidelines for the treatment of malaria to consider the timely inclusion of ACTs as a first-line therapeutic option for uncomplicated falciparum malaria, which was subsequently endorsed by WHO’s Technical Expert Group on Malaria Chemotherapy in December 2017 (Global Malaria Programme, 2015). Countries will then review the new WHO recommendation and make the decision whether to adopt the recommendation within national policy.

Broadly within malaria control, policy studies have documented and analysed barriers to policy making in East Africa (Paul *et al.*, 2015), the changing of first line treatment for malaria (Williams *et al.*, 2004), the non-normative process of adoption of larval source management (Tesfazghi *et al.*, 2015) and development of global policies for intermittent preventive treatment in infants (IPTi) and seasonal malaria chemoprevention (SMC) (D’Souza and Parkhurst, 2018). In control of MiP, studies have explored evidence for, and delays in policy change (Crawley *et al.*, 2007), documented the adoption of the revised IPTp-SP policy across countries of SSA (Henry *et al.*, 2018) but have not interrogated the policy adoption process and influences on this process.

We conducted a retrospective study of the policy adoption process at the national level for the revised global IPTp-SP policy (revised in October 2012) (World Health Organization, 2012) and a prospective policy adoption study for a future policy on treatment of first trimester malaria with ACTs (WHO Malaria Policy Advisory Committee, 2016a).

## Methods

### Study sites

The study was undertaken in Kenya, Mali, Malawi and The Gambia between February 2017 and February 2018. The epidemiology of malaria varies across the four countries. Kenya has four major malaria epidemiological zones: endemic, highland epidemic, semi-arid and seasonal and low risk, determined mainly by altitude, rainfall patterns and temperature; in Mali, malaria is endemic in the central and southern regions (where >90% of the population lives), epidemic in the north and highly seasonal; in Malawi, malaria is intense across the country with local variation in intensity and in The Gambia malaria transmission is heterogenous across the country (very low to low prevalence in western and central Gambia and moderate prevalence in eastern Gambia), and highly seasonal.

### MiP policies and timelines

**Figure 1:**
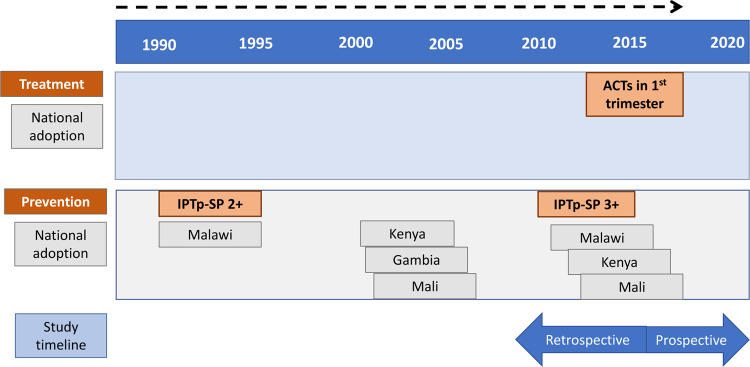
Timeline of WHO policies and recommendations for treatment and prevention of MiP in relation to study

The timelines for global and national policy adoption for each policy are provided in Figure 1, which includes the retrospective and prospective periods of the study in relation to IPTp-SP and ACTs, respectively. The policy on treatment of first trimester malaria with ACTs (WHO Malaria Policy Advisory Committee, 2016a) was under review by the WHO Malaria Chemotherapy TEG and was not adopted in any of the countries at the time of the study. The rationale for studying both of these policies simultaneously was that it provided the opportunity to use lessons learned from the retrospective IPTp-SP policy adoption to support and manage the prospective first trimester malaria treatment policy adoption (Walt *et al.*, 2008).

### Analytical framework

**Figure 2:**
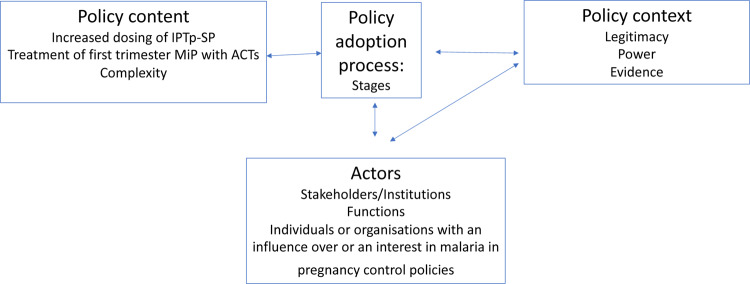
Analytical framework adapted from Walt (1994) and Tesfazghi *et al.* (2015)

An analytical framework was developed (Figure 2) to explore what happened and why with adoption of the revised IPTp-SP policy, and what is likely to happen with adoption of the policy on first trimester treatment of MiP and why (Walt *et al.*, 2008). Our focus was the adoption of WHO global policy to national policy. We defined adoption as the national decision-making process for the policy change in the country. The analytical framework provided a guide to our study tool development and analysis. Our framework was adapted from the policy triangle framework of Walt and Gilson (Walt and Gilson, 1994) including policy content, policy context, policy process, actors and power; and Tesfazghi *et al.* (2015) who described the functions of actors in the normative process of policy adoption for vector control in Nigeria. In our adapted framework, policy content is the technical content of the policy; process are the stages of the adoption process; context is not just the wider distal social, political and economic influences but more proximal factors within the realist concept of context as ‘the characteristics of the conditions in which the interventions are introduced’ (Pawson and Tilley 1997); and actors are individuals or institutions involved in or with an influence on policy decision-making. Our concept of context thus included perceptions of stakeholders on policy legitimacy, evidence and power.

Driven by our aim to understand the policy processes and actors we extrapolated from the functions of actors suggested by Tesfazghi *et al.* (2015) to describe four stages of the policy adoption process: Stage 1: identify a [policy] need; Stage 2: review the evidence; Stage 3: consult stakeholders and Stage 4: endorse and draft policy. We recognize that these stages may not in practice follow one after the other in a linear fashion (Sabatier, 2007) and that they may occur more than once. However, defining these stages aided our exploration of how different institutions and individual actors interacted within the policy adoption process, what roles they took, and what factors influenced their decision-making within these roles.

We assumed that perceived legitimacy, credibility and salience were important in the policy adoption of IPTp-SP in our study countries and were likely to be important for first trimester treatment of malaria with ACTs as was shown by D’Souza and Parkhurst recently in the global development process of two malaria control policies (D’Souza and Parkhurst, 2018). These policies were IPTi and intermittent preventive treatment of children under 5 years of age, which is now re-named as SMC. Legitimacy is the perception that evidence generation and use was unbiased; credibility that the evidence was of sufficient strength and quality and salience that the evidence was relevant to the needs of the decision-makers (Cash *et al.*, 2003). We were particularly interested in the capacity that stakeholders had to act and direct or influence the decision-making process of policy adoption and examined this through a power lens (Sriram *et al.*, 2018).

### Document review

A document review of published and unpublished national documents was first undertaken. Documents reviewed included: organograms and structures of ministries of health; national malaria policies, strategies and guidelines; national reproductive health policies, strategies and guidelines and President’s Malaria Initiative (PMI) operational plans (Table 1). Documents were primarily accessed at the national level through national level contacts among the authors and requests to stakeholders and were supplemented by online searches for peer-reviewed publications on malaria and MiP policies in PubMed. Information was extracted from the documents into an excel spreadsheet on the policy making context, the content of malaria and reproductive health policy documents, and the key stakeholders involved. This information was used in the selection of stakeholders, development of the theme guides and analysis of the interview data.

**Table 1:** Documents reviewed by category

Country	Category and document
	Structure of MoH and organograms
Kenya	Republic of Kenya National Malaria Control Programme: malaria fact sheet http://www.nmcp.or.ke/index.php/about-us
Malawi	Ministry of Health. 2011. *Malawi Health Sector Strategic Plan 2011–2016*. Lilongwe.
Mali	1. Programme Nationale de Lutte Contre le Paludisme. *Politique Nationale de Lutte Contre le Paludisme au Mali*. Bamako (Undated document). 2. Programme Nationale de Lutte Contre le Paludisme. 2013. *Plan National de Suivi/Evaluation 2013–2017*.
The Gambia	The Gambia: Ministry of Health and Social Welfare. 2012. *National Health Policy ‘Health is Wealth’ 2012–2020*. Banjul.
	National malaria policies, strategies and guidelines
Kenya	1. National Malaria Control Programme. 2014. *Kenya Malaria Strategy 2009–2018 (Revised 2014).* Nairobi. 2. Ministry of Public Health and Sanitation and Ministry of Medical Services. 2010. *National Guidelines for the Diagnosis, Treatment and Prevention of Malaria in Kenya*. Nairobi. 3. Republic of Kenya National Malaria Control Programme: http://www.nmcp.or.ke/index.php/resource-centre/download-centre/case-managment 4. Ministry of Health Division of Malaria Control. 2005. Malaria communication strategy. 5. National Malaria Control Programme (NMCP), Kenya National Bureau of Statistics (KNBS) and ICF International. 2016. Kenya Malaria Indicator Survey 2015. Nairobi, Kenya and Rockville, Maryland, USA: NMCP, KNBS and ICF International.
Malawi	1. National Malaria Control Programme. *Malaria Strategic Plan 2011–2015 Towards Universal Access*. Lilongwe. 2. National Malaria Control Programme. 2011. *Revised Guide for the Management of Malaria*. Lilongwe. 3. National Statistical Office (NSO) [Malawi] and ICF International. 2016. *Malawi Demographic and Health Survey 2015–16: Key Indicators Report*. Zomba, Malawi and Rockville, Maryland, USA: NSO and ICF International.
Mali	1. Programme Nationale de Lutte Contre le Paludisme. 2017*. Plan Strategique de Lutte Contre le Paludisme 2013–2017.* 2. Programme Nationale de Lutte Contre le Paludisme. *Politique Nationale de Lutte Contre le Paludisme au Mali*. Bamako (there are no dates for this document). 3. Programme Nationale de Lutte Contre le Paludisme. 2013. *Plan National de Suivi/Evaluation 2013–2017*. 4. Cellule de Planification et de Statistique du Secteur Sante Developpement Social et Promotion de la Famille. Canevas de synthese des rapports d’evaluation 2015 et de programmation 2017 du programme national de lute contre le paludisme.
**The Gambia**	1. National Malaria Control Programme. *National Malaria Strategic Plan 2014–2020*. 2. Ministry of Health and Social Welfare. 2009. *Malaria Control in The Gambia Strategic Plan 2008–2015*. Banjul. 3. The Gambia Bureau of Statistics (GBOS) and ICF International. 2014. *The Gambia Demographic and Health Survey 2013.* Banjul, The Gambia and Rockville, Maryland, USA: GBOS and ICF International. 4. National Malaria Control Programme. 2015. *The Gambia National Monitoring and Evaluation Plan for Malaria* 2014–2020.
	National reproductive health policies, strategies and guidelines
Kenya	1. Ministry of Health. 2007. *National Reproductive Health Policy: Enhancing Reproductive Health Status for all Kenyans*. Nairobi. 2. Division of Reproductive Health and National AIDS and STI Control Programme, Kenya. *National Curriculum on Sexuality and Sexual Health Training for Health Service Providers: Facilitator’s Manual*. June 2011.
Malawi	1. Ministry of Health. 2006. *National Reproductive Health Strategy 2006–2010*. 2. Ministry of Health. 2012. *Road Map for Accelerating the reduction of Maternal and Neonatal Mortality and Morbidity in Malawi*. Lilongwe. 3. Ministry of Health. 2009. *National Sexual and Reproductive Health and Rights (SRHR) Policy*. Lilongwe.
Mali	Ministere de la Santé & Programme National de Lutte Contre le Paludisme. 2012. Directives nationales pour la gestion et la distribution gratuite des moustiquaires impregnées d’insecticide de longue duree aux femmes enceintes et aux enfants de moins de cinq ans et de la sulfadoxine pyrimethamine á la femme enceinte.
The Gambia	1. Department of State for Health. *National Reproductive Health Policy 2007–2014*. 2. Sundby J. 2014. A rollercoaster of policy shifts: global trends and reproductive health policy in The Gambia. *Global Public Health* 9: 894–909. 3. Ministry of Women’s Affairs. *The Gambia National Gender Policy 2010–2020*. Banjul.
	PMI operational plans
Kenya	Kenya: President’s Malaria Initiative (PMI). Kenya Malaria Operational Plan FY 2016.
Malawi	Malawi: President’s Malaria Initiative (PMI). Malawi Malaria Operational Plan FY 2016.
Mali	Mali: President’s Malaria Initiative (PMI). Mali Malaria Operational Plan FY 2016.

### Selection of stakeholders

Stakeholders were purposively selected to include individuals and institutions with an interest in either the revised IPTp-SP policy and/or the first trimester treatment of MiP policy, who were affected by these policies or who, because of their position had or could have an active or passive influence on the decision-making and implementation processes (Varvasovszky and Brugha, 2000). The document review formed the starting point for the identification of key individuals and institutions and was supplemented following discussion with the study lead in each country. The final list for each country was formulated at a stakeholder selection session, where lists were compared and adjustments made. The final list broadly included stakeholders from: Ministry of Health (MoH) or other national institutions; National Malaria Control Programmes (NMCPs), National Reproductive Health Programmes (NRHPs); United Nations (UN) or bilateral institutions; Non-governmental Organisations (NGOs); international donors and academia. An iterative process was applied whereby additional stakeholders identified as relevant during the interviews were approached and invited to take part in the study.

### Data collection

In-depth interviews were conducted in: Nairobi, Kenya; Bamako, Mali; Blantyre and Lilongwe, Malawi and Banjul, The Gambia. Stakeholders were given information about the study and their written consent to be interviewed obtained. Interviews were then conducted by a trained social scientist in each country partner research institution using a theme guide. The theme guide related specifically to revised IPTp-SP policy (World Health Organization, 2012) and the first trimester treatment of MiP recommendation (WHO Malaria Policy Advisory Committee, 2016b), and was standard across the four countries and included: formal policy making structures; perceptions of policy legitimacy; prospective or retrospective support or opposition from competing interest groups; perceived fit within the health system of the intervention; anticipated future benefits and costs. However, an iterative process was applied to the interviews, such that additional themes were included and further interrogated as they arose during the interviews. These new themes were then included in subsequent interviews.

Interviews were conducted in English except for Bamako where they were conducted in French. Data collected during stakeholder interviews was digitally recorded and supplemented by handwritten field notes. Data were transcribed, and translated (Bamako), and imported into NVivo (QSR International) Version 11 for data management and analysis.

### Data analysis

A coding framework was constructed where the primary coding nodes were based on the elements of our analytical framework. This deductive coding was supplemented by inductive coding based on additional themes that emerged from the data using content analysis (Bernard, 2006). Coding was conducted by one author but analysed and interpreted by three authors to enhance objectivity.

For each stage of policy adoption the process and actors with a specific role were identified. Study participants were assigned an anonymous code during data analysis which related to their category of stakeholder (see below), and these were used to label quotes to ensure anonymity. This approach was followed for both the retrospective and prospective studies.

The Standards of Reporting Qualitative Research (SRQR) which provides clear standards for reporting through a list of 21 essential items was used to guide the reporting of findings (O’Brien *et al.*, 2014). The SRQR does not specifically include an analytical framework within its reporting guidance on qualitative approach, but does include guiding theory more broadly.

## Results

A total of 47 interviews were completed with stakeholders across the four countries (Table 2). The results are presented according to each of the analytical framework themes: policy content for both the revised IPTp-SP (World Health Organization, 2012) and the prospective treatment of first trimester malaria with ACT policies (WHO Malaria Policy Advisory Committee, 2016a): the policy processes, roles and actors for each of the four stages of the policy adoption process using examples from the adoption of revised IPTp-SP policy; and policy context. Context included the presentation of stakeholder perceptions of policy legitimacy, stakeholder power, followed by presentation of perceptions of the revised IPTp-SP policy (World Health Organization, 2012) and the treatment of first trimester malaria. Findings from the document review are presented and cited where relevant.

**Table 2:** Interviewees by institution and country

Institution	Kenya	Malawi	Mali	The Gambia	Total
MoH, other national institution	3	3	4	3	13
NMCP	3	4	6	1	14
Reproductive health programme (NRHP)/other national programme	2	1	1	0	4
UN, bilateral	3	0	2	2	7
NGO	3	1	2	1	7
International donor	0	0	0	1	1
Academia	0	1	0	0	1
	14	10	15	8	47

### Policy content

#### IPTp-SP policy

IPTp-SP has been national policy for prevention of MiP in Kenya, Malawi, Mali and The Gambia for more than two decades ( , 2017, 2018a,b,c) with policies stating at least two or at least three doses to be given to pregnant women at least 1 month apart starting in the second trimester (Programme Nationale de Lutte Contre le Paludisme; Ministry of Public Health and Sanitation and Ministry of Medical Services, 2010; National Malaria Control Programme, 2011; van Eijk *et al.*, 2011). The study countries have updated this policy within the last 5 years to reflect the WHO revised policy on IPTp-SP (National Malaria Control Programme, 2014, 2015; National Malaria Control Programme. Ministry of Health and Social Welfare, 2014) Each country has stated that IPTp-SP should be given to pregnant women at least 1 month apart on each visit or each scheduled visit to antenatal clinic (ANC).

#### Treatment of first trimester malaria policy

In all study countries, quinine is currently recommended for the treatment of malaria in pregnant women during the first trimester, and ACT in the second and third trimesters (Ministry of Public Health and Sanitation and Ministry of Medical Services, 2010; National Malaria Control Programme, 2011; National Malaria Control Programme; Programme Nationale de Lutte Contre le Paludisme, 2013). Combining quinine with clindamycin supports the move away from monotherapies. Based on the reports of stakeholders, there was an almost universal lack of adherence to this current policy on treatment for first trimester MiP (Hill *et al.*, 2014; Riley *et al.*, 2016). Clindamycin was reported not to have been procured in Kenya, Malawi and The Gambia. ACTs are not entirely contraindicated for first trimester treatment; for example, the Kenyan policy on treatment of first trimester MiP states that artemether–lumefantrine (AL) must not be withheld if quinine is not available (Ministry of Health, 2016) and in Malawi, ACTs are recommended for first trimester women with confirmed treatment failure to quinine plus clindamycin (Ministry of Health, 2015). The prospective policy currently under discussion at WHO is the replacement of quinine with ACTs for the treatment of first trimester malaria.

### Policy process, roles and actors

Interviews with stakeholders initially focused on general health policy processes, followed by the process through which the number of doses of IPTp-SP in the revised policy was changed. Comments were then sought on potential issues relating to the anticipated announcement of a global policy shift on the treatment of malaria in first trimester pregnant women from quinine to ACTs. Policy changes for malaria control were reportedly led by the NMCP in all four countries. In addition, stakeholders across all countries reported that policy changes involving MiP required close collaboration with NRHPs.

#### Stage one: identify a policy need

Policy needs were identified through global initiatives based on WHO recommendations, international standards and successful pilots at global level, through advocates, activists or government departments. Whilst the IPTp-SP dosing change was a recommendation of WHO, awareness of stakeholders within countries and therefore the route to initial identification of a need varied. In Kenya it was based on the 2012 revised WHO recommendations on IPTp-SP (World Health Organization, 2012) and subsequently the NMCP decided that changing IPTp-SP dosing was a priority. In The Gambia, the dosing changes were a recommendation of the 2012 WHO Malaria Programme Review and were followed by forums where focal points from the NMCP discussed the rationale of the change. In Malawi, it was considered a simple process to adapt the guidance from WHO and no further evidence generation or syntheses were undertaken. In Mali, key stakeholders taking part in international meetings were aware of other countries moving to 3 doses following which, consultation between the NMCP and the Malaria Research and Training Centre (MRTC) led to the policy process being initiated.

**Figure 3:**
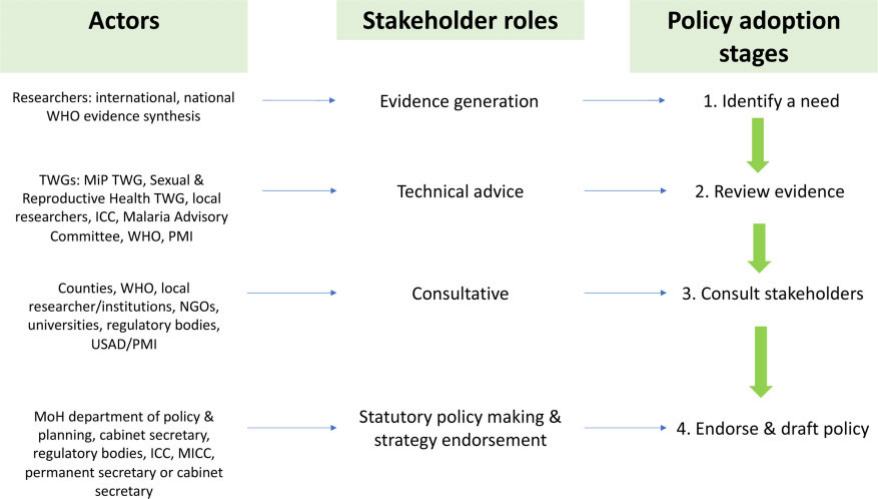
Stages of the policy adoption process, actors and their role

Ultimately a need should be identified through evidence, whether internationally or locally driven. Evidence generation was reported to be done mainly by researchers, both international and local, including the Kenya Medical Research Institute (KEMRI) (Kenya), College of Medicine (Malawi) and MRTC (Mali) (Figure 3). Researchers from MRTC led the review of evidence that led to the change in international recommendations on IPTp-SP dosing (Kayentao *et al.*, 2013).

#### Stage two: review the evidence

Each of the four countries reported the presence of national technical working groups (TWGs) with the role of reviewing malaria evidence for policy. In Kenya, Malawi and Mali TWGs were established groups with regular meetings, whilst in The Gambia, they were *ad hoc* task forces established as needed. Where the evidence was external to the country options were to: do a pilot or operational research study in-country to generate locally derived evidence; or review the evidence and consider whether it reflects the country situation, is implementable and likely to give the expected results. Often both options were reported as having been conducted concurrently. The nature of the evidence and who had produced or was recommending the evidence was felt to be important. It was felt that if WHO were recommending a malaria policy, then the studies were sufficient and further studies would build on this evidence. For example, in Malawi the evidence on the policy of using rapid diagnostic tests (RDTs) to diagnose malaria was accepted based on WHO evidence and then national studies were undertaken to test different types and brands of RDTs. In Kenya, the availability of local evidence on use of ACT for treatment of severe malaria meant that the policy was adopted quickly. However, lack of local evidence led to the MRTC decision in Mali to stall the adoption of ACT use in second trimester until it could be gathered, as they felt the international evidence did not reflect the Malian context.

All countries reported that the evidence for changing IPTp-SP dosing had been reviewed by their TWGs and that supporting this policy was straight forward, that is their decision-making was not complicated because the evidence was available and had been reviewed and endorsed by WHO.

A MiP TWG was mentioned by stakeholders in Kenya, Malawi and Mali, together with a Sexual and Reproductive Health (SRH) TWG in Malawi. The TWGs generally involved a wide range of experts and stakeholders. In Malawi and Kenya, the MiP TWG is chaired by a member of National Reproductive Health Programme, with the secretariat in the NMCP. In Mali, the MiP TWG is chaired by NMCP, with the Reproductive Health Programme amongst the members.

#### Stage three: consult stakeholders

The critical importance of stakeholder consultation in defining and drafting policies was stated by participants in all four countries. In Kenya, it was the role of the NMCP to bring together partners to consider the feasibility when deciding whether to adopt malaria policies, and in Mali, for MiP, the NMCP had a similar role but under the umbrella of the TWG. Consultative partners reported that only two meetings were needed in The Gambia to bring about the change in IPTp-SP dosing.

Consultation on the revised IPTp-SP policy being developed involved stakeholders from the MoH and development partners as well as those involved in implementing this intervention. Representatives from the Counties or Districts for example, districts health officers, were often included. In The Gambia, the revised IPTp-SP policy development in malaria control was reported to have included stakeholder consultation on the strengths, weaknesses, opportunities and threats of the new policy.

#### Stage four: draft policy, statutory policy making and strategy endorsement

In most countries, standards and policy procedures are reviewed every 5 years. If the required update is close to official revision time then countries will wait and include it, otherwise they make an addendum or technical note while waiting for official date of policy review. In the case of these MiP policies the relevant national documents are the National Malaria Strategic Plan and the National Malaria Treatment Guidelines. Sometimes, as reported by The Gambia, consultants are hired to facilitate the consultation meetings and help draft the policy.

The analysis of revising the IPTp-SP policy across the four countries revealed it was often an advisory committee that made a final decision on the IPTp-SP policy, that is, endorsed the policy. The definition of this committee varied across the four countries. In Kenya, TWGs present recommendations to the Malaria Inter-Agency Coordinating Committee (MICC) for endorsement. Endorsement includes consideration of whether the recommendation reflects the country situation, is implementable and likely to produce expected results. The Principal Secretary, MoH in Kenya is chair of the MICC. A similar body in Malawi is the National Malaria Advisory committee which meets only twice a year. Once a policy has been endorsed, it is signed by a senior member of the Ministry of Health.

### Policy context

#### Policy legitimacy

Stakeholder perceptions of the legitimacy of a new or revised policy will contribute to a positive context for adoption of the policy. When asked what made a policy legitimate, participants across the four countries reported the following key criteria, which aligned with the four stages of the policy adoption process: (1) the policy was endorsed by the appropriate authority, which usually meant the relevant ministry within government, (2) the policy was based on WHO recommendations, (3) the formulation of the policy had involved key stakeholders and (4) the policy was based on evidence. Additional factors described by stakeholders in some countries included that the policy should address a need ‘on the ground’, improve health, be feasible to implement and be acceptable to the public.

#### Stakeholder power

Stakeholders in all four countries strongly identified with the bureaucratic power of the leadership role of the WHO in guiding recommendations on MiP control policies (Sriram *et al.*, 2018). UN and bilateral donors were particularly strong in their expression of the leadership role of the WHO:

*Respondent (R): WHO is the truth. So WHO says it has to happen, it has to happen …* (Kenya_UN/bilateral).

*R: If WHO already approves it is no longer worth doing operational studies* (Mali_UN/bilateral).

Some stakeholders were aware of the WHO ERG and MPAC processes in the development of WHO policy guidelines. The role of the national research institutions and TWGs in reviewing the evidence from WHO was valued in all countries, however, in The Gambia external support was sometimes called upon.

The policy adoption process for any MiP control strategies was routinely led by NMCP in all four countries with the support of NRHP. Each country, with the exception of The Gambia, had a MiP TWG which was chaired by either NMCP or NRHP.

The guidance of national research institutions in reviewing international malaria control policies for potential adoption was said to be particularly strong in Mali, indicating a power based on technical expertise (Sriram *et al.*, 2018). It was suggested by a stakeholder that MRTC could question WHO guidance if local evidence was not judged to be sufficient. For example, the change in policy on the use of ACTs in the second and third trimesters was delayed in Mali as MRTC were not comfortable that the study data was representative of the Malian population, as most of the studies used to support the change were conducted in Asia. The relationship between the NMCP and MRTC in Mali was very strong, with the MRTC being described as the ‘technical arm’ of NMCP.

Despite the defined structures and processes for malaria policy making, stakeholders remarked on the political pressures experienced within the process, such as political pressure on the WHO from donors at the international level, and from pharmaceutical companies at the national level. In Malawi, a knowledge translation unit was developed within the MoH to strengthen the use of evidence for decision-making, but some participants felt that platform was understaffed and often side-lined during the policy making process.

### Evidence

#### Revised intermittent preventive treatment policy

The IPTp-SP dosing policy change was perceived to have been backed by strong evidence and endorsed by the WHO. As such, the decision of whether to revise this policy was reported to have been an easy one.

*R: It was quite easy because evidence was there that giving three, four doses is more beneficial than limiting the dose to two, so as a result it was an easy process* (The Gambia_MoH/other government body).

The major challenge in increasing the doses of IPTp-SP reported by stakeholders related to perceptions of the feasibility of implementing the policy. Many stakeholders, across all four countries, expressed doubt that high coverage of the new dosing schedule could be achieved as coverage targets for the previous schedule, which included fewer doses, were not yet met.

#### First trimester treatment

We now present the perceptions of stakeholders on the current and potential future policies on first trimester treatment for MiP.

##### Current policy.

Compliance to quinine for the treatment of first trimester MiP was reported as poor across the four countries both in terms of health worker prescribing practices and women’s adherence to the treatment. Health workers sometimes prescribed ACTs, despite contravening current policy guidance, due to poor quinine tolerance among women and a dislike of the bitter taste.

*R: The only challenge that we get with this policy is that …….from the health workers, yes they have the information but at times they would still be able to prescribe AL Just because of the mother is being able to tolerate. Yeah, so there is less compliance when it comes to quinine vis a vis AL and you will get that the health workers at times just go ahead and give the AL* (Kenya_NRHP).

Perceptions on resistance to quinine varied with some stakeholders believing that resistance was already a problem and others feeling sure that there was no resistance to quinine, with no clear differences across the countries.

In Mali, some stakeholders said they would welcome a shift from quinine to ACTs for treatment in the first trimester as some health providers were unsure whether to use quinine or ACTs in the first trimester due to their inability to accurately determine gestational age. The switch to ACTs would simplify the treatment guidelines, particularly in areas where community health workers (CHWs) are delivering services. CHWs are not currently authorized to prescribe ACTs due to the contraindication in first trimester. A second concern expressed by the TWG was that by treating pregnant women in the community, ANC attendance would fall.

*R: I think there are concerns; the first difficulty is the age of pregnancy, whether it is the first trimester of pregnancy, whether or not we should give ACTs. The gestational age is a serious problem with our providers especially in CSCOM* [Community Health Centres] *where it is the matrons who are in charge of that, it is true, there are clinical signs but it can’t be said with confidence …* (Mali_UN/Bilateral).

##### Potential future policy.

Participants listed their perceptions of many benefits of ACTs including ACTs are very effective antimalarials; are more readily available; combination therapies are better than monotherapies; have fewer side effects than quinine; have shorter dosing schedule and fewer compliance issues.

*R: Midwifes said that pregnant women are always complaining about quinine because of its adverse effects …. and nurses don’t want to do the prescription because of side effect. Often the number of doses is reduced to avoid side effects. The treatment is always not correctly done. Women take just few tablets for few days. Even if the parasitaemia is reduced, the time of treatment is not enough and this can lead to severe malaria* (Mali_NMCP).

Concerns were expressed that there may be resistance to ACTs as there have been reports of treatment failures.

Most stakeholders did not have a preference for a specific ACT, amongst those who did – AL was mentioned based on efficacy studies (Malawi) and dihydroartemisinin–piperaquine (DP), or that selection depended on the safety profile of the specific antimalarial and the evidence. Several stakeholders reported that they would worry about using amodiaquine (Malawi and Mali) due to its impact on the liver.

*R: I would have to look at the scientific evidence that is available, but I know that what we are usually worried about is the partner drug. The ACTs come with fast acting drug and long acting drugs. Fast acting will be artesunate or artemisinin derivative and usually there is very little or not at all safety concerns because it is not available for a long time … but we have artemether-lumefantrine, piperaquine and some use SP and amodiaquine, those are long acting drugs … amodiaquine that is the one I would be a little bit hesitant … but you have to look at the evidence* (NRHP/other national programme).

##### Evidence required to support a change from current to future policy for first trimester treatment of malaria.

Perceptions on the evidence required to introduce ACTs for treatment in the first trimester in the future focused on safety and efficacy. The main safety concern was teratogenic effects as well as other adverse reactions, with no recognition that data on adverse effects was from animal models only. The need to carefully balance the potential risks of the drug with the benefits in terms of improved outcomes of MiP was acknowledged. Stakeholders from Malawi and The Gambia felt that the evidence from ‘WHO studies’ was not yet conclusive and they were awaiting an official announcement from WHO. There were concerns from stakeholders in Malawi that WHO guidance would be based on systematic reviews that included studies from outside the region and rather should be based on empirical studies from neighbouring countries, and from Kenya that local evidence of safety and efficacy was required, not just global evidence. Several stakeholders mentioned that there must have been a reason for non-use of ACTs in the first trimester, having been deemed unsafe initially, and that pharmacovigilance should be in place for this policy change.

*R: With artesunate there is a problem since in the first trimester and in safety studies artesunate has shown that it is teratogenic and embryotoxic. But there is no evidence in terms of research. Because there is no evidence the assumption is that we can proceed to artesunate since it has better efficacy than quinine. But I think…change to artesunate in the second and third trimester when that risk of teratogenic and embryotoxicity is gone* (Malawi_MoH/national institution).

Participants drew parallels on their experience of changing policy for first line treatment of malaria from SP to ACTs based on national drug resistance studies and guidance from WHO on ‘threshold’ levels of SP resistance at which a change should be initiated. It was acknowledged that lessons on tolerability could be learned from other countries already using the drug.

### Stakeholder position on first trimester treatment with ACTs

Most participants felt there would not be any opposition to a future policy change to ACT for first trimester treatment and could not name an organization that would oppose it. Most participants reported that if the evidence was good and the recommendation came from WHO and was endorsed by the NMCP then the policy would be supported by the wider development community and other stakeholder groups.

However, responses on potential opposition to a future policy change to ACT for first trimester treatment related mainly to evidence being viewed as unsatisfactory. In Kenya it was felt that studies from civil society showing evidence counter to the recommendation could potentially be a risk, although no such studies had been reported thus far. Other potential triggers of opposition included critics in the scientific community (Mali), medical practitioners if they are not convinced of safety of the drugs (Kenya, Malawi), and potential challenges from the private sector (The Gambia).

## Discussion

In this study, covering a timeline from 2012 to 2018, we investigated the perceptions of national level stakeholders on the adoption of two global malaria control policies into national level policies in four SSA countries; the 2012 revised IPTp-SP policy (World Health Organization, 2012) (using a retrospective lens) and a potential future policy change on the use of ACTs for first trimester treatment of MiP (WHO Malaria Policy Advisory Committee, 2016a).

The policy adoption process for changing the IPTp-SP dosing regimen was very similar across the study countries, each using the four stage policy adoption process extrapolated from functions of actors in Tesfazghi *et al.* (2015) including: identification of policy need, review of evidence, stakeholder consultation and drafting policy/policy endorsement. The prompt for initiating the policy change process in all countries was the recommendation from WHO (WHO Global Malaria Programme, 2013). Stakeholders reported that the WHO endorsement was a requirement for the legitimacy of a malaria control policy, however not all policies recommended by WHO are adopted by countries as was the case with IPTi (D’Souza and Parkhurst, 2018).

There is a large and growing literature on the use of research evidence in policy making including models of the way in which the evidence is used and the process within which it is used (Gilson *et al.*, 2018). Policy formulation at the global level for the revised IPTp-SP policy (World Health Organization, 2012) was a top-down model primarily focused on research evidence from a systematic review (Kayentao et al., 2013). The policy adoption process at the national level in each of the four countries however, followed an interactive research utilization model where the research evidence was part of a wider process involving a range of stakeholders, with participation of stakeholders perceived as a requirement for the policy to be legitimate (Buse *et al.*, 2012).

The revised IPTp-SP WHO recommendation (World Health Organization, 2012) was perceived to be based on good quality, credible evidence. The evidence was perceived as relevant as the included studies were all from SSA and covered West, East and Southern Africa regions: Burkina Faso, Kenya, Malawi, Mali, Tanzania and Zambia, hence there was no requirement for additional local evidence. This evidence also underwent a high level of peer review by a WHO ERG before being passed to the MPAC for further review and endorsement. None of the stakeholders interviewed across the four countries perceived there to have been any national opposition to this policy, nor any deviation from the normative policy change process. The evidence generation and review process were seen as legitimate.

The policy process for malaria control interventions is not always this straightforward, particularly but not only where the evidence base is not strong and widely accepted. Long delays were experienced in changing from chloroquine to SP as first line malaria treatment policy in SSA despite strong evidence (Williams *et al.*, 2004) at a time when drug policy change processes were less formulated and institutionalized. In other cases, the normative process had not been followed due to strong stakeholder interests and perceptions, and stakeholder power. For example, the policy decision on scaling up larviciding in Nigeria deviated from the normative process, involving powerful actors and a commercial incentive, and excluding those usually involved in evidence generation, advisory and consultative roles (Tesfazghi *et al.*, 2015). Consensus for other malaria control policies has struggled despite the evidence being considered credible. A recent policy study found that the evidence for SMC was generated from randomized control trials (RCTs) in the seasonal transmission settings in which it would be applied and results were similar. Conversely, the RCTs for IPTi were done in a variety of transmission settings as IPTi is an intervention that can be implemented throughout the year. These different transmission and drug resistance settings led to less consistent results in the trials and therefore a less consistent efficacy message (D’Souza and Parkhurst, 2018). This study highlights the differing perceptions of the legitimacy of the process of SMC compared with that of IPTi, partly not only due to the type of evidence but also due to the policy structures, e.g. MPAC was in place for SMC but not IPTi, and lessons learned from IPTi, but also the perceived advocacy for IPTi from technical actors.

Interviews with stakeholders across the four countries suggest that the future adoption of treatment in first trimester with ACTs would be less straight forward than for changing the IPTp-SP regimen. A future first trimester treatment policy change would involve more actors such as drug regulators given this is a new indication for ACTs and an off-label use, as the producers do not currently recommend their use in the first trimester. The evidence for use of ACTs in the first trimester was perceived to be less strong and therefore less convincing, particularly on safety, which was considered paramount by all actors. Respondents noted that whilst the meta-analysis which led to the change in IPTp-SP policy was based on findings from RCTs in a number of countries, the evidence for the adoption of ACTs for first trimester MiP was from a meta-analysis of observational studies. However, RCTs to assess the efficacy of ACTs vs quinine in the first trimester of pregnancy have not been conducted as they have been considered unethical, and the sample size required to provide any guidance on the risk of congenital abnormalities prohibitive (Dellicour *et al.*, 2017). Concerns were expressed on the representativeness of the population in which the first trimester safety studies were conducted. Despite the meta-analysis containing six studies from SSA, the title of the publication indicated that the analyses were based on data from Africa and Asia and this may have been taken at face value. Although adverse events from ACTs may be similar in Africa and Asia, the differences in the nature of clinical malaria in the two continents, due to differing transmission levels, are widely known. Participants felt pharmacovigilance studies at the country level, and the support to conduct them, would be required (Talisuna *et al.*, 2006; Dellicour *et al.*, 2007, 2008; Tinto *et al.*, 2015). Prospective pharmacovigilance studies are the only way to increase the evidence base for safety in the first trimester to capture first trimester exposures, especially in the first few weeks of pregnancy, when women often do not know that they are pregnant or do not admit to their pregnancy.

Whilst many stakeholders expressed reservations about the credibility and salience of some MiP evidence, others had sufficient trust in the WHO and the legitimacy of the global processes and would have confidence in a future national policy change to ACTs for treatment in the first trimester if/when recommended by WHO. For some participants, the need for local evidence was paramount. Stakeholder consultation and the reaching of consensus in each country would require strong leadership. In Mali, given the dynamic between the NMCP and MRTC, MRTC support for the policy change was important.

Our examination and description of the policy adoption stages, functions and identity of stakeholders within this process will be useful when interrogating and navigating the policy process for other malaria control interventions. Application of our simple analytical framework highlighted the strong influence, in national adoption of global policy, of the perceived applicability or salience of evidence to the country not just credibility or quality of evidence, which resonates with the findings of D’Souza and Parkhurst in their study on IPTi and SMC (D’Souza and Parkhurst, 2018). Malaria and/or MiP TWGs had a strong role in decision-making on national adoption of global policies. The *two communities theory* of research utilization proposes that the research and policy communities operate within different cultures (Caplan, 1979). However, for countries in this study, the malaria and MiP TWGs bring researchers and decision-makers together to make joint recommendations. Whilst we were able to gain an understanding of the wider policy making structures within our study countries, there would be value in further exploration of the decision-making processes, relationships and power within such national TWGs.

Policy analysis is often limited by lack of access to clear documentation in terms of content and across programmes and departments (Walt *et al.*, 2008; Gomez *et al.*, 2014). In this study, there was a lack of clarity on policy dates. The study was also limited by the availability of potential interviewees. Despite the study team’s best efforts to interview all selected interviewees some key stakeholders were not available. This challenge accounted to a large extent for the difference in numbers of stakeholders interviewed between the countries. There was confusion in some interviews with stakeholders on the national policy process for updating national policy guidelines, for example the national malaria strategy update, often a 5-year cycle vs new drug regimens or changes to a specific intervention.

## Conclusions

National policy adoption of three or more doses of IPTp-SP was seen to be based on strong evidence, a recommendation from WHO, consensus from stakeholders, and followed the normative process for updating an existing policy. A future change from quinine to ACTs for first trimester treatment of MiP is likely to be supported due to the poor tolerability of quinine compared to ACTs and because it would simplify provider practice. However, concerns about safety in the first trimester and lack of evidence at the country level will need to be addressed to garner full support for national policy adoption. Strong national leadership will be needed to reach stakeholder consensus and policy adoption.

## Funding

The study was funded by EDCTP as part of the EDCTP2 programme supported by the European Union [CSA-MI-2014-276 IMPPACT). The funding body had no role in the design of the study and collection, analysis and interpretation of data and in writing the manuscript.

Conflict of interest statement. None declared.

*Ethical approval.* Ethics approval was obtained from the Kenya Medical Research Institute Scientific and Ethics Review Unit (Kenya), College of Medicine Research and Ethics Committee (Malawi), Ethical Committee of Faculté de Médecine et d’Odonto-Stomatologie et Faculté de Pharmacie, University of Sciences, Techniques and Technologies of Bamako (Mali), The Gambian Government/Medical Research Council Unit the Gambia Joint Ethics Committee (The Gambia), the Ethics Committees of the London School of Hygiene and Tropical Medicine and the Liverpool School of Tropical Medicine. Respondents were informed of the details of the study and written consent sought before interviewing.
